# Supplementary material for: Efficacy and Biomarker Analysis of Adavosertib in Differentiated Thyroid Cancer
Source: Cancers (Basel). 2021 Jul 12;13(14):3487. doi: 10.3390/cancers13143487 (PMC8306685; doi:10.3390/cancers13143487)
Supplement: Supplementary file 1 [file cancers-13-03487-s001.zip › cancers-1251349-supplementary.pdf]

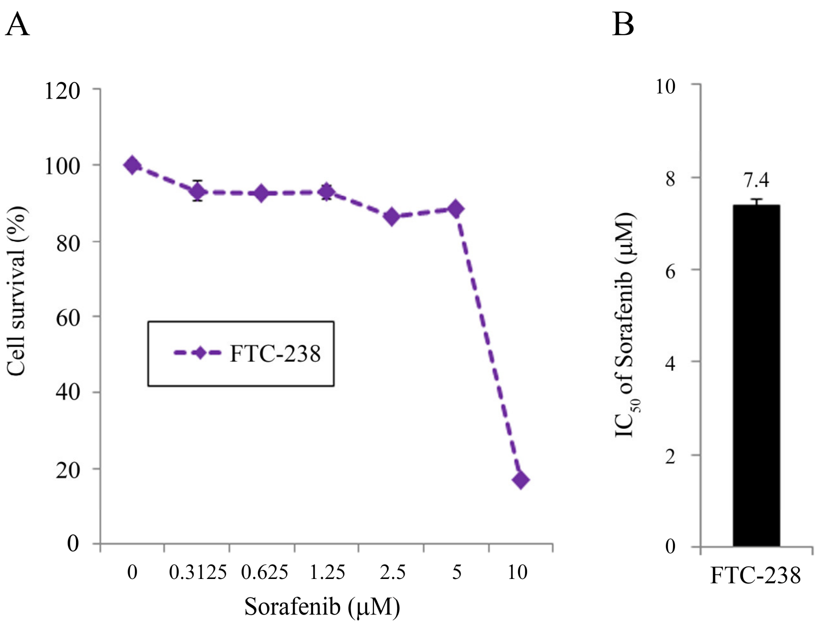

Figure 1. Sorafenib treatment reduced cell viability in the FTC-238 cell line. (A) LDH assay was performed to determine cell viability by sorafenib treatment in FTC-238 cells for a 4-day treatment course. Dose-response curve was obtained. (B) The median-effect dose (IC_{50}) of sorafenib in FTC-238 cells was determined by CompuSyn software.

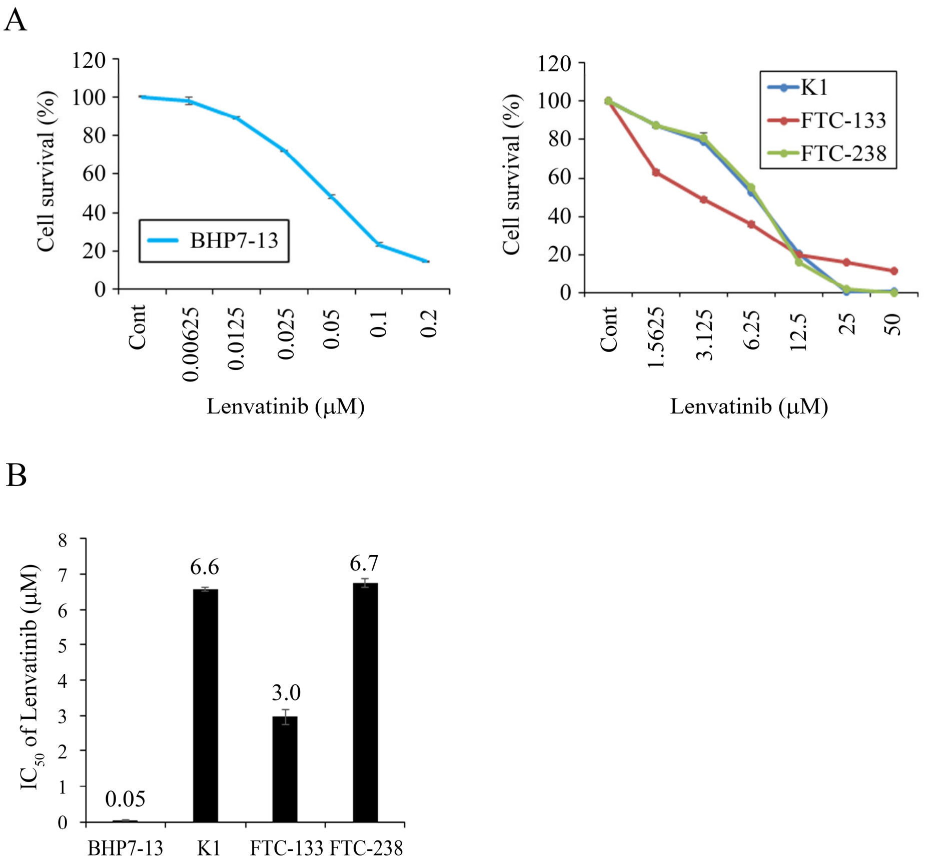

Figure 2. Lenvatinib treatment reduced cell viability in 4 DTC cell lines. (A) We performed a lactate dehydrogenase assay to determine cell viability after lenvatinib treatment in BHP7-13, K1, FTC-133, and FTC-238 cells for a 4-day treatment course and obtained a dose-response curve. (B) The median-effect dose (IC_{50}) of lenvatinib in the 4 DTC cell lines was determined by CompuSyn software.

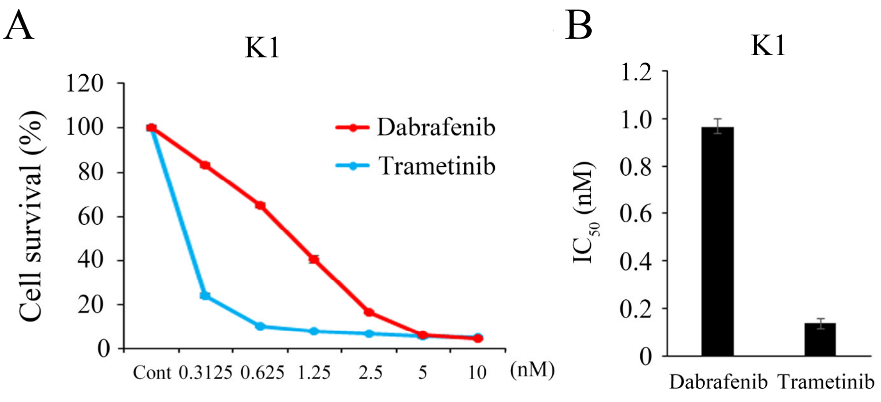

Figure 3. Dabrafenib and trametinib treatment reduce cell viability in K1 cells. (A) Lactate dehydrogenase assays were performed to determine cell viability after dabrafenib and trametinib treatment in K1 cells for a 4-day treatment course. Dose-response curves were obtained for dabrafenib and trametinib, respectively. (B) The IC_{50} profiles of dabrafenib and trametinib in K1 cells were determined by CompuSyn software.

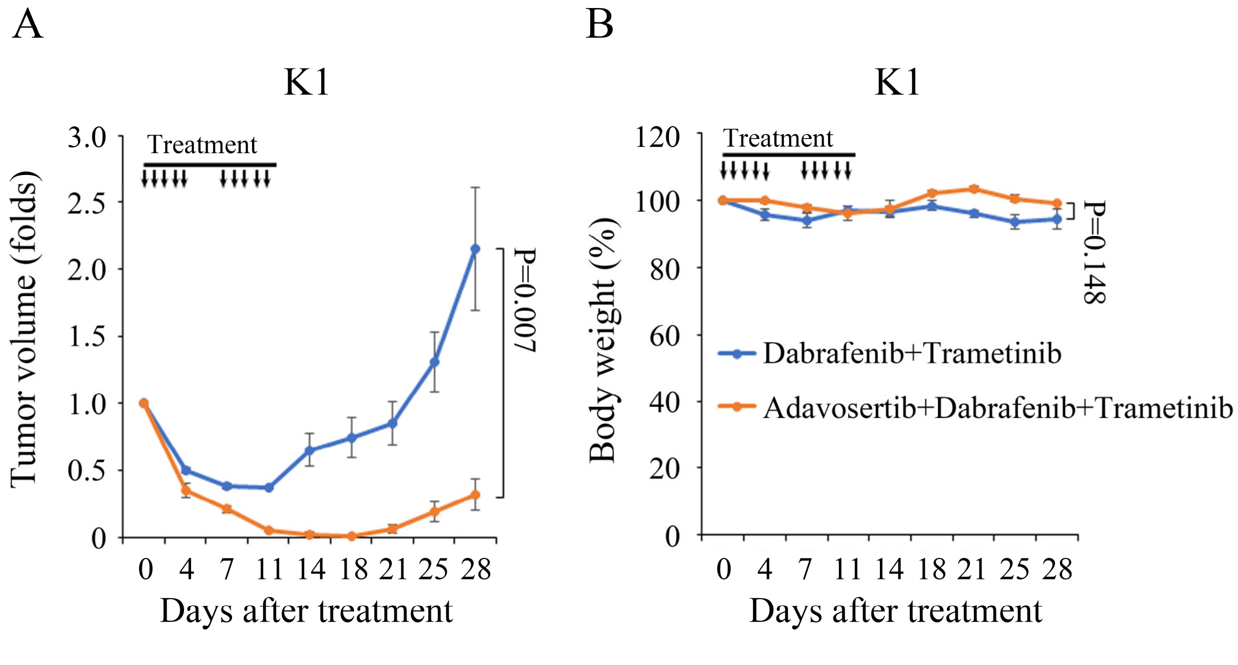

Figure 4. Triple combination therapy of adavosertib, dabrafenib, and trametinib robustly retards subcutaneous xenograft growth in a papillary thyroid cancer model. (A) Nude mice bearing K1 xenografts were treated with an oral gavage of dabrafenib (30 mg/kg) plus trametinib (0.6 mg/kg), or triple combination of adavosertib (50 mg/kg), dabrafenib (30 mg/kg), and trametinib (0.6 mg/kg) daily for two cycles of 5 days on and 2 days off. The triple combination of adavosertib, dabrafenib, and trametinib significantly inhibited K1 tumor growth when compared with dabrafenib and trametinib combination treatment ($p < 0.05$ for both comparisons between days 7 and 28). (B) Triple combination therapy did not significantly alter body weight compared with dual therapy.

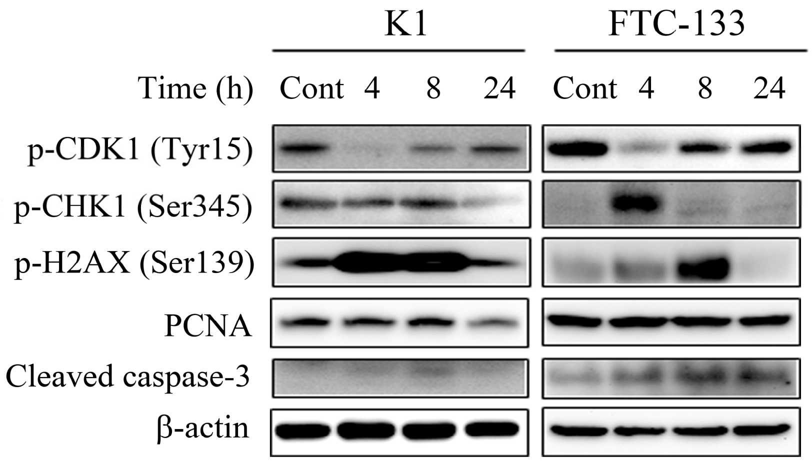

Figure 5. Immunoblot analysis of p-CDK1, p-CHK1, p-H2AX, PCNA, and cleaved caspase-3 expression in K1 and FTC-133 tumors treated with adavosertib. Tumor levels of p-CDK1 (Tyr15), p-CHK1 (Ser345), p-H2AX (Ser139), PCNA, and cleaved caspase-3 were evaluated using immunoblot in K1 and FTC-133 xenografts after a single oral dose of adavosertib (50 mg/kg).
