# Supplementary figures and images for: Efficacy and Biomarker Analysis of Adavosertib in Differentiated Thyroid Cancer
Source: Cancers (Basel). 2021 Jul 12;13(14):3487. doi: 10.3390/cancers13143487 (PMC8306685; doi:10.3390/cancers13143487)

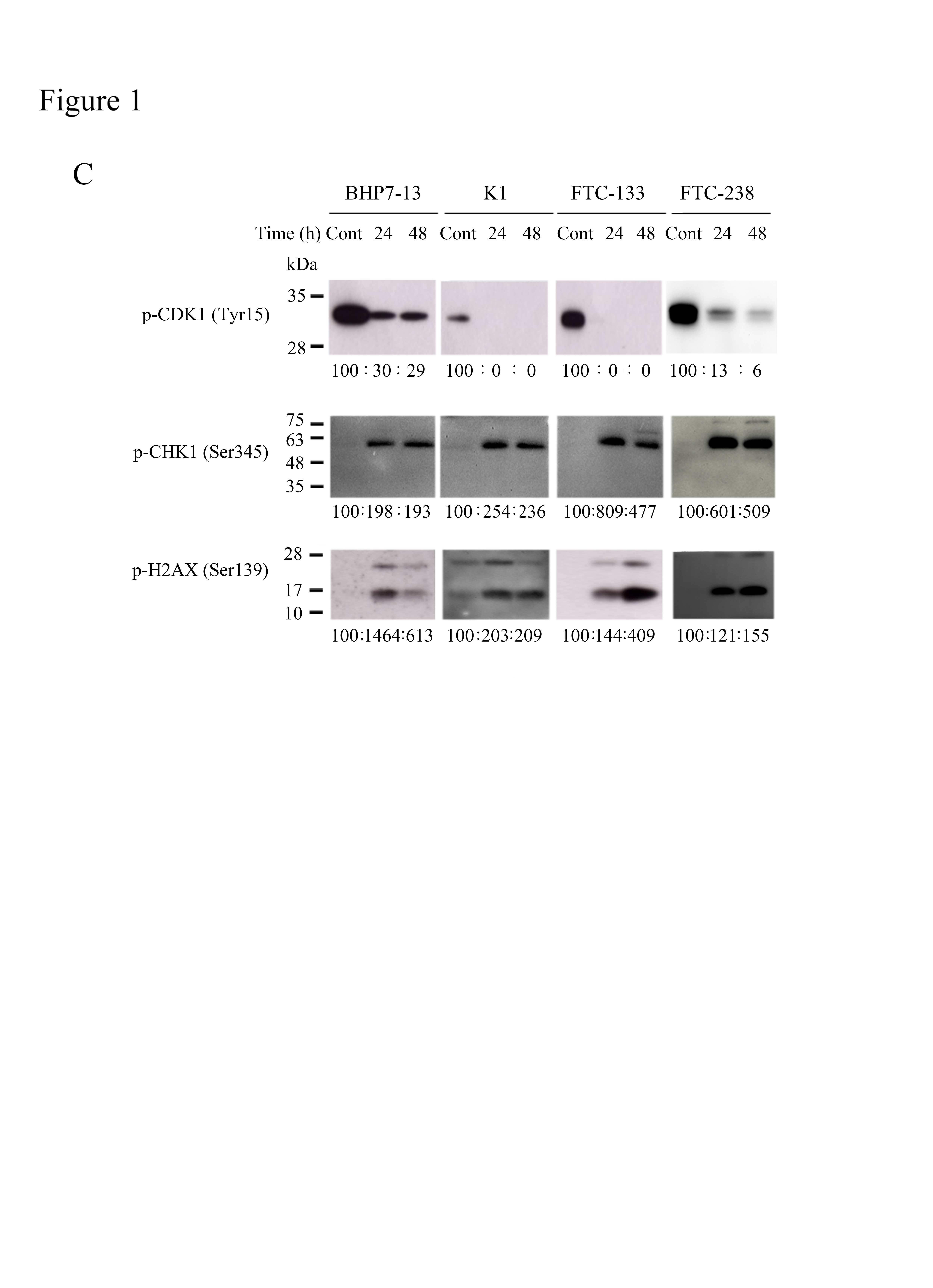

Supplement: Supplementary file 1 [file cancers-13-03487-s001.zip › whole WB figures/Fig 1C.tif]

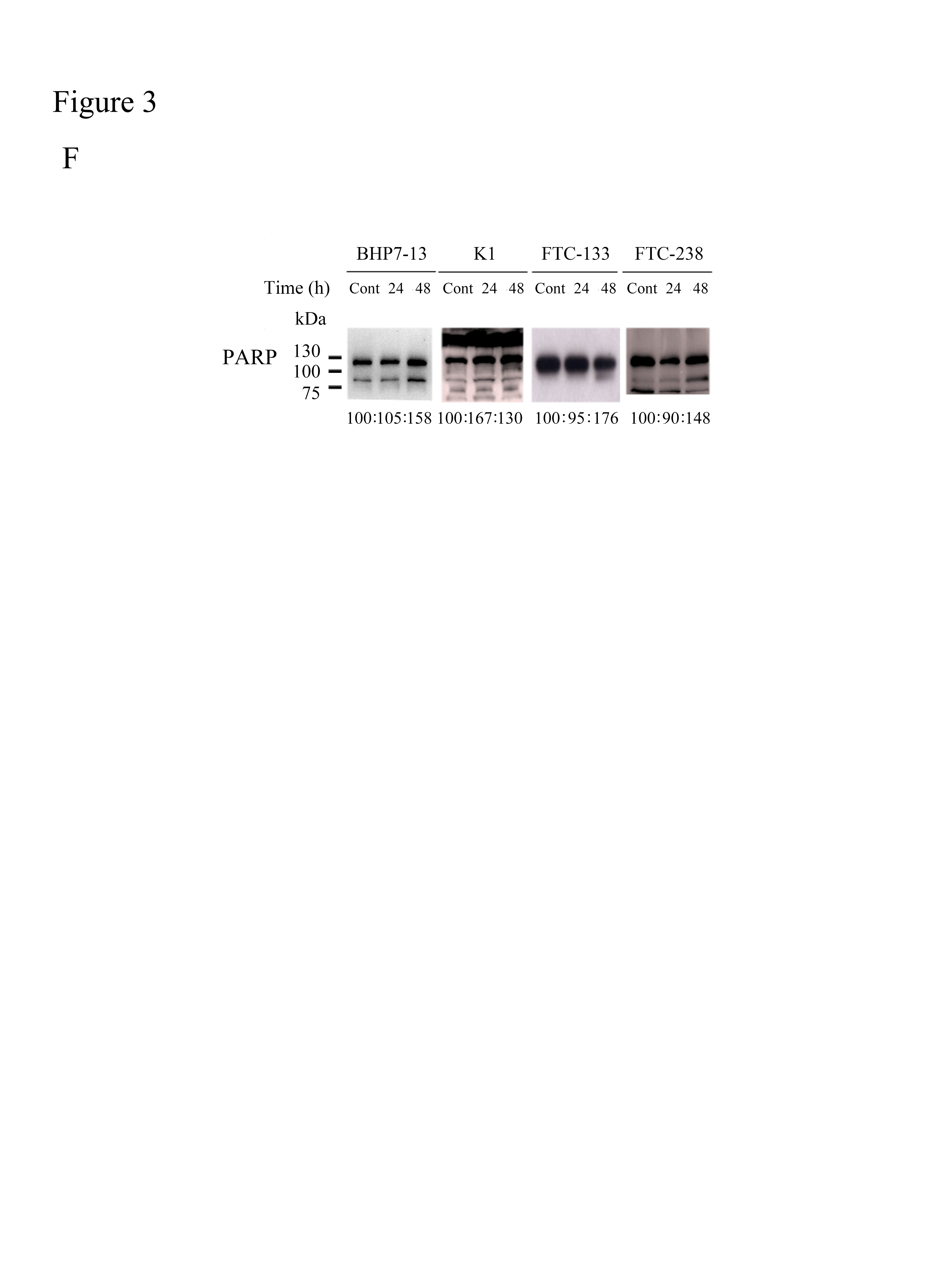

Supplement: Supplementary file 1 [file cancers-13-03487-s001.zip › whole WB figures/Fig 3F.tif]

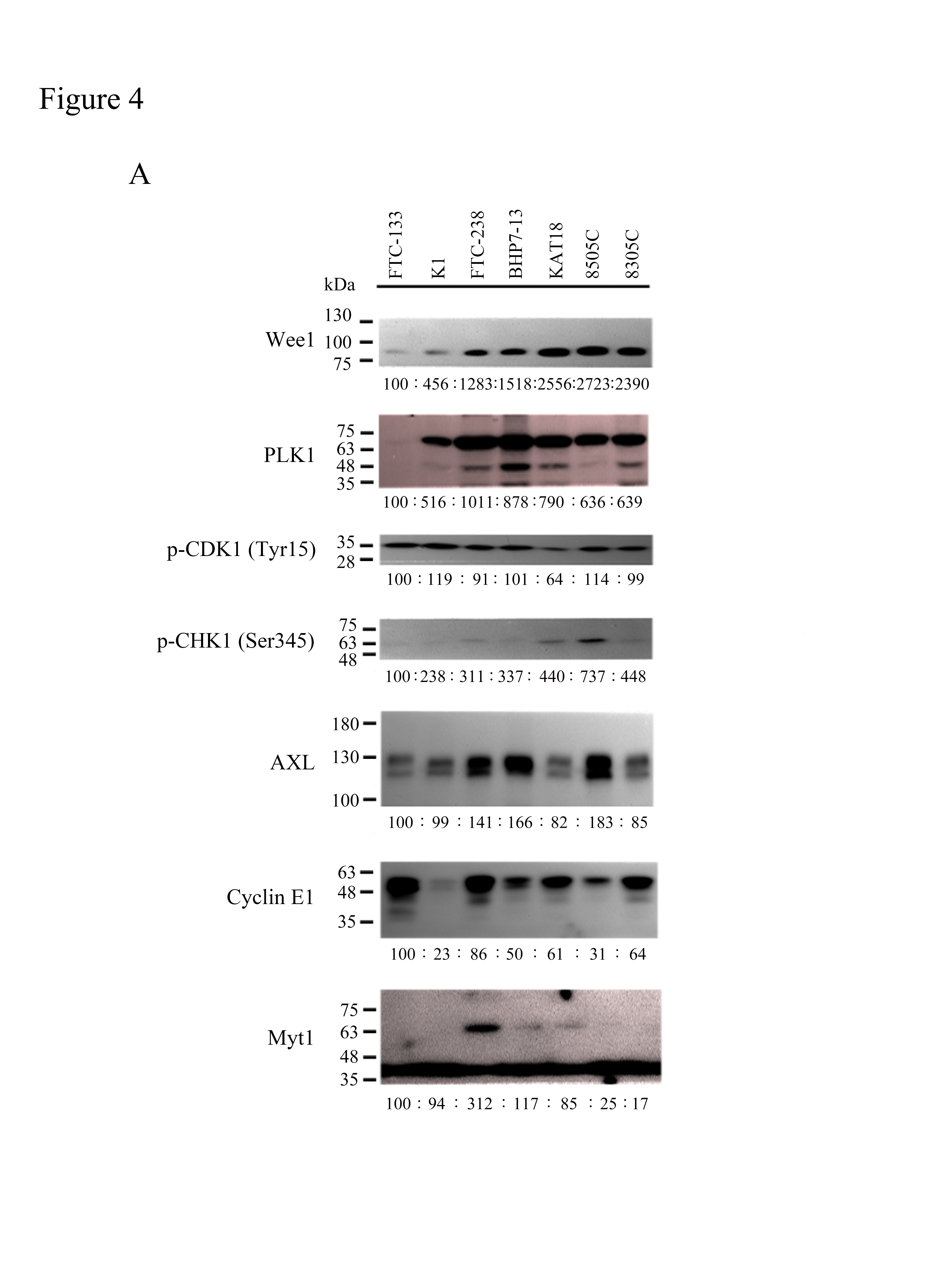

Supplement: Supplementary file 1 [file cancers-13-03487-s001.zip › whole WB figures/Fig 4A.tif]

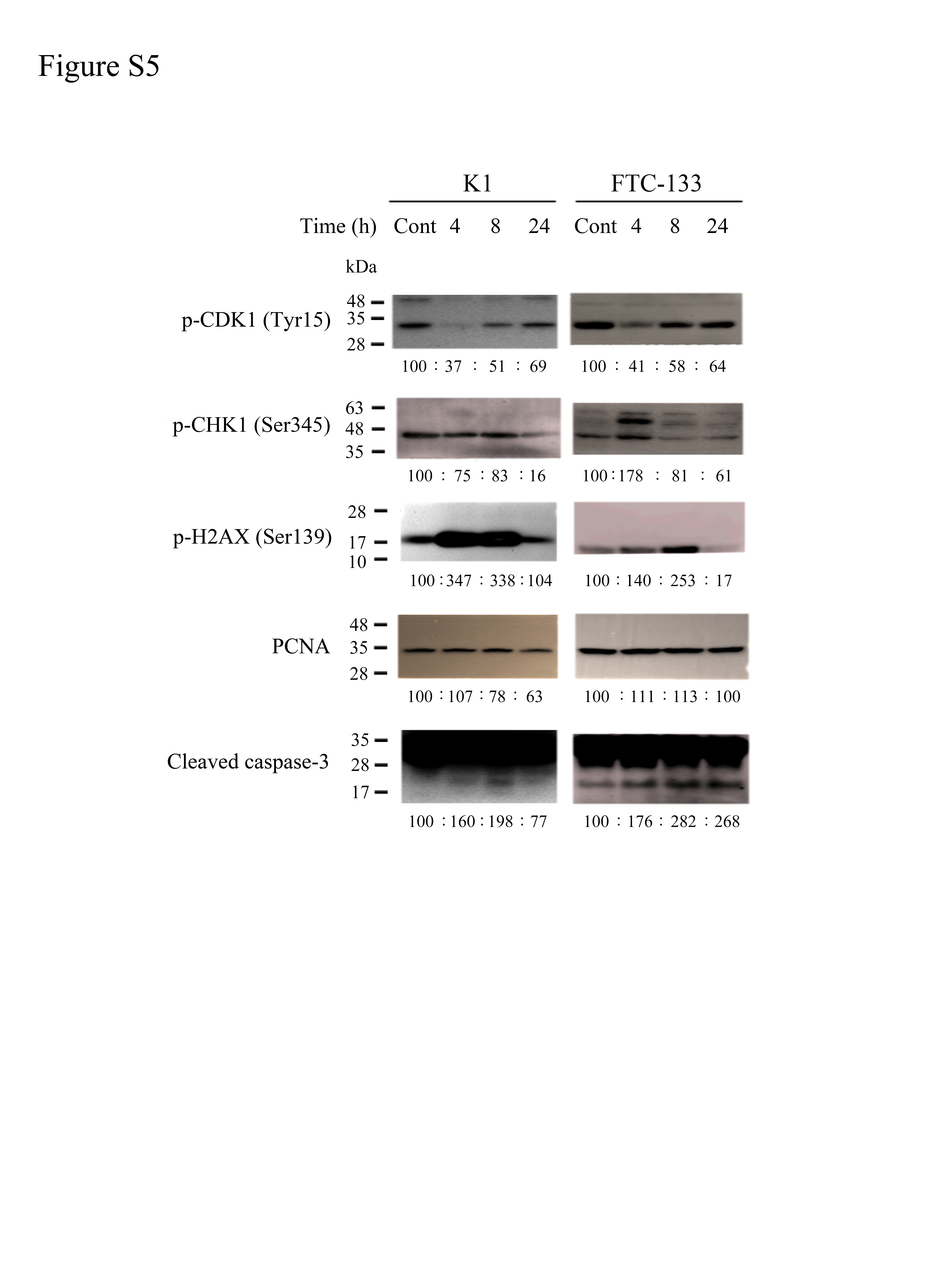

Supplement: Supplementary file 1 [file cancers-13-03487-s001.zip › whole WB figures/Fig S5.tif]
